# Supplementary material for: Cellulose Fibre Degradation in Cellulose/Steel Hybrid Geotextiles under Outdoor Weathering Conditions
Source: Polymers (Basel). 2022 Oct 5;14(19):4179. doi: 10.3390/polym14194179 (PMC9573017; doi:10.3390/polym14194179)
Supplement: Supplementary file 1 [file polymers-14-04179-s001.zip › polymers-1910437-supplementary.pdf]

Supplementary Information

Cellulose fibre degradation in cellulose/steel hybrid geotextiles under outdoor weathering conditions

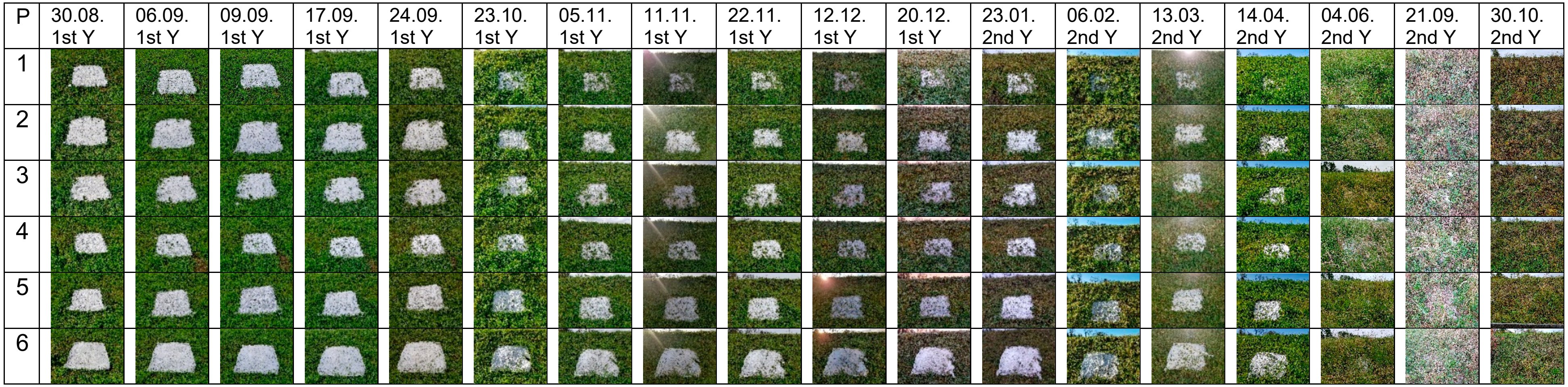

Figure S1. Photodocumentation of the biodegradation of the test prototypes P1 – P6 as function of test time.
